# Preparation and Thermo-Mechanical Characteristics of Composites Based on Epoxy Resin with Kaolinite and Clinoptilolite

**DOI:** 10.3390/polym15081898

**Published:** 2023-04-15

**Authors:** Andrzej Puszka, Marcin Kneć, Wojciech Franus, Beata Podkościelna

**Affiliations:** 1 Department of Polymer Chemistry, Institute of Chemical Sciences, Faculty of Chemistry, Maria Curie-Skłodowska University, M. Curie-Skłodowska Sq.3., 20-031 Lublin, Poland; andrzej.puszka@mail.umcs.pl; 2 Laboratory of Construction, Faculty of Civil Engineering and Architecture, Lublin University of Technology, 40 Nadbystrzycka St., 20-618 Lublin, Poland; m.knec@pollub.pl; 3 Department of Construction Materials Engineering and Geoengineering, Faculty of Civil Engineering and Architecture, Lublin University of Technology, 20-618 Lublin, Poland; w.franus@pollub.pl

**Keywords:** epoxy resin, crosslinking, thermal properties, natural fillers, mechanical properties

## Abstract

Herein the synthesis, characterization, and study of spectroscopic, thermal, and thermo-mechanical properties of polymeric composites are presented. The composites were obtained in special molds (8 × 10 cm) based on the commercially available epoxy resin Epidian^®^ 601 cross-linked by 10% *w*/*w* triethylenetetramine (TETA). To improve the thermal and mechanical properties of the synthetic epoxy resins, natural fillers in the form of minerals from the silicate cluster kaolinite (KA) or clinoptilolite (CL) were added to the composites. The structures of the materials obtained were confirmed by attenuated total reflectance-Fourier transform infrared spectroscopy (ATR/FTIR). The thermal properties of the resins were investigated by differential scanning calorimetry (DSC) and dynamic-mechanical analysis (DMA) in an inert atmosphere. The hardness of the crosslinked products was determined using the Shore D method. Moreover, strength tests were performed on the 3PB (three-point bending) specimen, with the analysis of tensile strains conducted using the Digital Image Correlation (DIC) technique.

## 1. Introduction

Epoxy compounds have been known since the late 19th century. Their development has progressed rapidly during this time. Today, epoxy-based plastics are used in many areas of human activity. Initially, they were substitutes for scarce natural materials and later became excellent replacements [1,2]. They are on a par with materials such as wood, metals, glass and ceramics, and many plastics surpass natural materials in their properties. Epoxy resins are brittle solids or viscous liquids that contain epoxy groups in their structure, which are capable of polyreactions that transform them into cross-linked, insoluble and infusible polymers. Cured resins are also referred to as epoxy resins, although they no longer contain epoxy groups [3,4,5,6].

The curing process is accompanied by fundamental changes in the physical properties of the resin-curing agent system. The curing reactions are exothermic and the heat released during the reaction process increases the temperature of the system in question so that the reaction between resin and hardener is accelerated. An undesirable temperature rise can be counteracted by adding the same amount of filler to the resin, which absorbs the heat given off. It is also possible to reduce the amount of hardener or to use a hardener with retarding additives [7,8,9,10,11].

Crosslinked epoxy resins are characterized by high abrasion, scratch and impact resistance. Other features are their compressive, crushing and tensile strength and high hardness. The properties of epoxy resins also include above-average resistance to moisture, high temperatures, oil and grease, and certain other chemicals (e.g., acids and salt solutions). These outstanding properties have resulted in epoxy products being the standard option for a variety of applications such as adhesives, coatings and composites for structural applications [1]. The main disadvantages of epoxy resins include lower resistance in contact with chlorine, methanol or hydrocarbons, as well as in exposure to ultraviolet (UV) light and scratching. Various compounds are added to epoxy resins to change and/or improve their properties. The most common are flame retardants, fillers, thickeners or pigments [12,13,14,15,16,17,18,19]. The use of various natural additives increases the eco-attractiveness of epoxy-based plastics and adapts their properties to specific applications.

Bekeshev et al. studied the effect of a polyfunctional modifier oligo (resorcinol phenyl phosphate) and a dispersed diorite (mineral filler) on the mechanical properties of epoxy-based composites. The functionalization of the diorite surface has been proven, and an increase in the strength characteristics of epoxy-based composite materials by 10–48% was obtained [20]. Bafakeeh et al. proposed two different kinds of hybrid fibers: woven carbon and glass fiber, while two different ceramic nanoparticles, alumina (Al_2_O_3_) and graphene nanoplatelets (GNPs), were chosen to incorporate into an epoxy resin [21]. Sim et al. described the preparation of a fly ash/epoxy composite using fly ash filler classified as industrial waste. The behavior of its mechanical properties was investigated. The fracture surfaces were characterized and correlated with the mechanical properties [22]. In turn, a comparative analysis of the influence of various finishing agents on the properties of epoxy compounds filled with potassium polytitanates was carried out by Burmistrov et al. [23].

Su et al. described the kaolinite/epoxy resin nanocomposites. The addition of modified kaolinite into the polymeric matrix improved the storage modulus and glass-transition temperature of the samples studied [24]. Fan et al. proposed the synthesis of composites based on epoxy resin with the multi-functional kaolinite-based hybrid to enhance flame retardancy, smoke suppression and anti-aging properties. The results showed the presence of only 3 wt% of hybrid can increase the value of the limiting oxygen index as well as decrease the value of the heat release rate compared to neat EP [25]. Hamidi and Marandi reported that cement and epoxy resin is used for the stabilization of soft clay soils. The results show that using epoxy resin increases strength parameters by about 100 to 1000 times [26]. 

The main aim of this work was the synthesis and thermal characterization of composites based on the commercially available resin Epidian^®^ 601 crosslinked with triethylenetetramine. As natural fillers, two minerals from the silicate cluster kaolinite or clinoptilolite were applied. Detailed spectroscopic and thermo-mechanical characterization has been presented. The influence of the percentage content of the additive on the strength properties in each of the tested groups was also determined using the DIC technique.

## 2. Materials and Methods

### 2.1. Chemicals

The epoxy resin Epidian^®^ 601 (EP601) was purchased from CIECH Sarzyna S.A. (Nowa Sarzyna, Poland). Epidian^®^ 601 is a clear, viscous liquid of light color, epoxy number: 0.500–0.550 mol/100 g, viscosity at 25 °C: 700–1100 mPas. The curing agent triethylenetetramine (TETA) was bought from Sigma-Aldrich (Darmstandt, Germany). Kaolinite with the molecular formula Al_2_O_3_·2SiO_2_·2H_2_O) was a commercial product purchased from Sigma-Aldrich (Darmstandt, Germany). Natural clinoptilolite (with typical molecular formula (Na_2_K_2_Ca)_3_Al_6_Si_30_O_72_·24H_2_O) came from the Nižný Hrabovec deposit (Slovakia).

### 2.2. Composite Preparation

**Figure 1:**
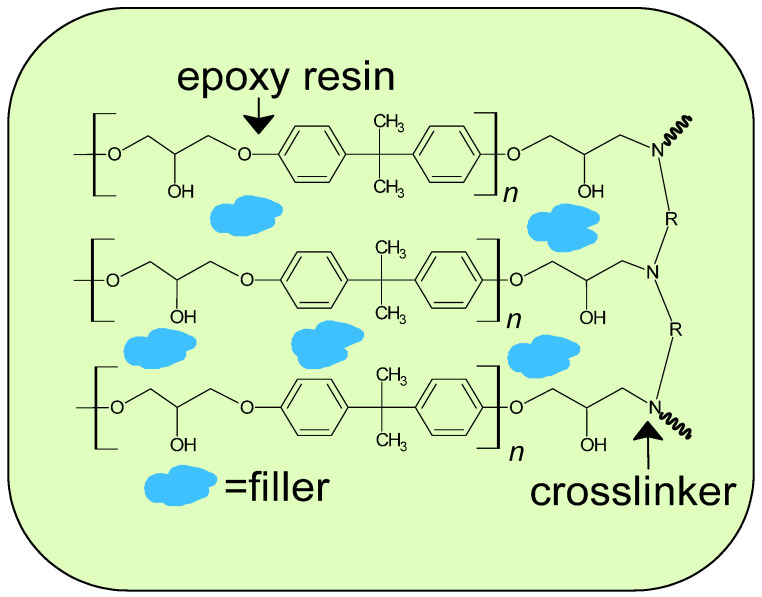
Proposed scheme of composite structure.

An appropriate amount of crosslinker (TETA) was added to the epoxy resin EP601 (Table 1). The chemicals were mixed at room temperature until a homogenous solution was obtained. Then the filler (kaolinite or clinoptilolite) was added in amounts of 1, 5, and 10 wt% to the weight of the epoxy resin + curing agent. The whole content was stirred and next put into the oven (for 10 min at 50 °C). The beaker contents were poured into glass molds (10 mm × 8 mm × 3 mm) and polymerized for 24 h at room temperature. The samples were heated at 70 °C for 60 min after being tested. Figure 1 presents the proposed scheme of the composite structure. 

A total of 7 plates with dimensions of approximately 50 mm × 90 mm were designated for bending tests, labeled and sequentially numbered as follows: CL5; KA1; CL10; 0 (reference material); KA5; CL1; KA10.

Complete sets of three or four samples with dimensions of approximately 80 mm × 12 mm × 3 mm were cut from the designated materials. Each sample was measured in terms of cross-section before testing. When four samples were cut for analysis, the three with the highest bending strength were selected.

### 2.3. Measurements Methods

#### 2.3.1. Fourier Transform Infrared Spectroscopy (FTIR)

The FTIR spectra were developed by applying attenuated total (internal) reflection (ATR/FTIR) with the use of a Bruker TENSOR 27 spectrophotometer (Ettlingen, Germany), equipped with a PIKE measuring cell which features crystalline diamond embedded in zinc selenide. The FTIR spectra were recorded within the range of 4000 to 600 cm^−1^, with 64 scans per sample, at a resolution of 2 cm^−1^ in the absorption mode. 

#### 2.3.2. Thermal and Thermomechanical Properties

##### Differential Scanning Calorimetry (DSC)

DSC thermograms were obtained using a Netzsch 204 F1 Phoenix calorimeter (Günzbung, Germany), by standard ISO 11357-1:2016 [27]. The sample of 10.0 ± 0.05 mg was weighed and was first cooled to an isotherm for 3 min at −100 °C and then heated up to a maximum temperature of 200 °C, next cooled to −100 °C and then heated to 200 °C. The scans were performed at the heating/cooling rate of 10 °C/min under an argon atmosphere (gas flow = 30 cm^3^/min). All DSC measurements were taken in aluminum pans with a pierced lid (a mass of 40 ± 1 mg). As a reference, an empty aluminum crucible was applied. The reported transitions were taken from the first and second heating scans. Glass-transition temperatures (T*_g_*_s_) for the polymer samples were taken as the inflection point on the curves of the heat-capacity changes. Curing temperatures (T*_cur_*_s_) were read at exothermic-peak maxima.

##### Dynamic Mechanical Analysis (DMA)

DMA of composites was performed in tensile mode using a DMA Q800 Analyzer TA Instruments (New Castle, DE, USA). Calibration was performed as per the manufacturer’s recommendations included in Advantage Software, version 5.5.24 (TA Instruments, New Castle, DE, USA). The experiments were carried out on rectangular samples of dimensions close to 3 mm thick, 10 mm wide, and 60 mm long. Experimental conditions employed were a frequency of 1 Hz and amplitude of 10 μm with the scanning air temperature range from −100 °C to 127 °C and a temperature ramp of 3 °C/min. The variations of storage modulus (*E′*), loss modulus (*E″*), and tangent delta (tan δ) versus temperature were determined.

#### 2.3.3. Mechanical Properties

The hardness of the composites was measured by the Shore D method on a Zwick 7206/H04 hardness tester (Ulm, Germany). The readings were taken after 15 s at a temperature of 23 °C [28]. Bending strength was measured using a Zwick 2.5 kN static machine. Pairs of images to calculate major and minor strain directions were recorded by an ARAMIS SRX, Zeiss (Jena, Germany) DIC system. The cross-sectional area of the sample along with the support span and force information was used to determine the bending strength according to Formula (1):

(1) $$\sigma =\frac{3Fl}{2bh^{2}}\left[\mathrm{MPa}\right]$$

where: *σ*—bending stress, *F*—maximum force, *l*—support span, *b*—sample width, *h*—sample height.

The force signal from the Zwick machine was connected to the ARAMIS SRX, Zeiss (Jena, Germany) system controller. This made it possible to relate the tensile strain values to the force values.

**Figure 2:**
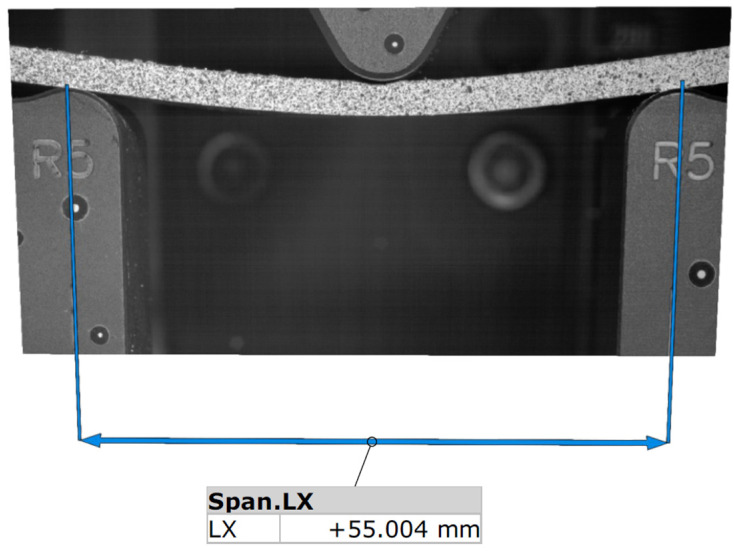
The measuring plan setup with span length.

Figure 2 shows the measurement plan view from the software that operates the ARAMIS SRX system. 

**Figure 3:**
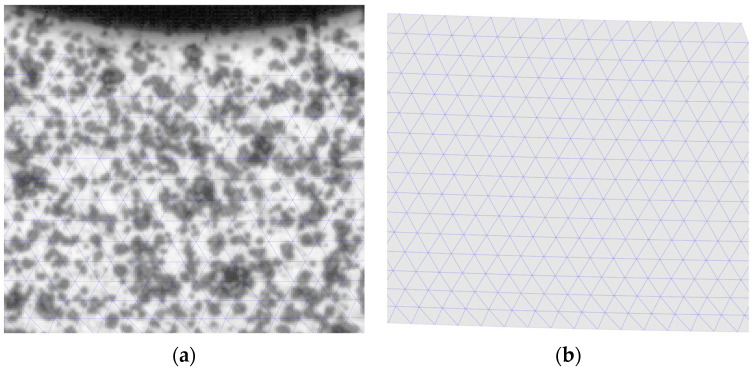
(**a**) Random pattern and applied grid; (**b**) Measurement grid “tied” to the sample surface.

Measurements of geometry, displacement, and strain can be performed in the software. Strain measurements are performed using the DIC technique, which allows for the creation of measurement grids (Figure 3b) that are “tied” to the zone on which they are created. Correlation is possible by overlaying a small stochastic pattern of black and white points on the sample (Figure 3a) and dividing the whole into small subregions called facets.

**Figure 4:**
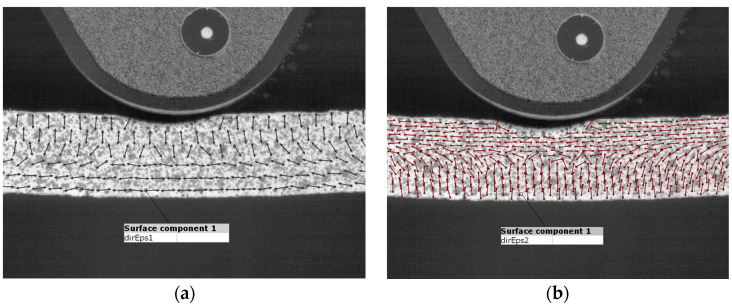
Principal deformation directions (**a**) major and (**b**) minor.

The tests were carried out at a constant speed of 3 mm/min. Figure 4 shows the map of the principal deformation directions of the major strains (Eps1) and minor strains (Eps2) on the sample during bending. The Eps1 directions visualize where the material carries the largest strains and in which directions. It can be seen that the lower layers of the sample carry the strains horizontally in the X direction determined in the analyses. These layers are stretched. Similarly (Figure 4b) shows which layers are compressed. As in any pure bending test, there is a so-called neutral layer in the center of the cross-section that does not carry any strains.

#### 2.3.4. Scanning Electron Microscope (SEM)

A Quanta 250 FEG (Field Emission Gun) scanning electron microscope (SEM) manufactured by FEI (Hillsboro, OR, USA) was used to investigate the morphology of the structure of the composite.

## 3. Results and Discussion

The crosslinking reaction of the epoxy resin Epidian 601 using TETA resulted in rigid polymeric composites. To fully crosslink the systems, the samples were kept at 70 °C for 1 h in a heating chamber. The reaction was carried out in special molds and then samples were cut for mechanical testing. Six systems differing in the amount of natural filler (kaolinite (KA) or clinoptilolite (CL)) were obtained. A sample without filler was also obtained and taken as the reference sample.

### 3.1. Spectroscopic Characteristics

**Figure 5:**
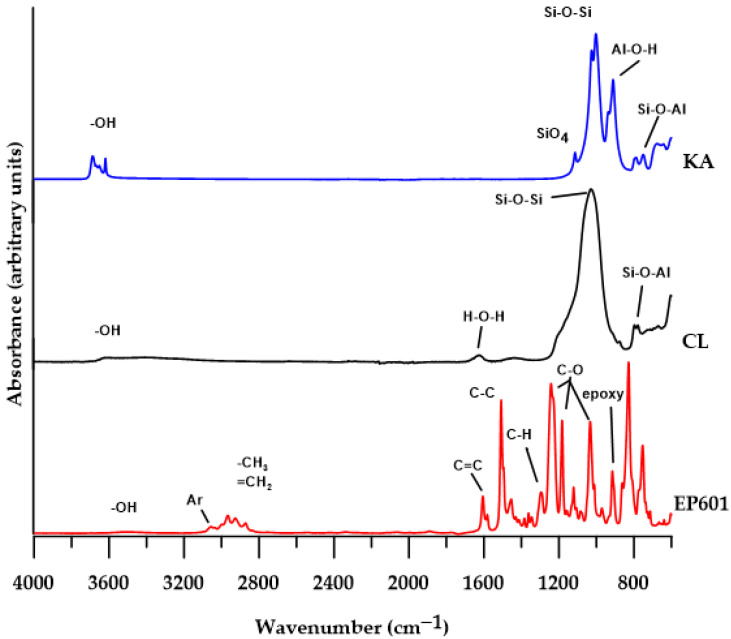
ATR/FTIR spectra of pure EP601 and fillers: CL and KA.

Characterization of chemical structure by the spectroscopic analysis ATR/FTIR was carried out for all materials obtained before and after DMA analysis. Figure 5 shows the spectra of the pure EP601 and pure fillers. 

The ATR/FTIR spectra of KA show characteristic absorption bands associated with vibrations: 3616–3686 cm^−1^—vibrations of OH groups in the crystal structure, 1114 cm^−1^—stretching vibrations of the tetragonal structure of SiO_4_; 1026 and 1003 cm^−1^—stretching vibrations of Si-O-Si; 911 cm^−1^—Al-O-H bending vibrations of the Al_2_OH group; 788, 750 and 676 cm^−1^—stretching vibrations of Si-O, Si-O-Al as well as Al-O-H [29].

The ATR/FTIR CL spectrum shows that there is water in its structure (absorption bands at 3398 and 3615 cm^−1^ characteristic of the OH group vibrations and a band at 1620 cm^−1^ corresponding to the H-O-H stretching vibrations of the water molecule). In addition, the spectrum shows the absorption bands at 1029 cm^−1^—stretching vibrations of Si-O and Al-O, and the absorption bands at 797, 779 cm^−1^—stretching vibrations of Si-O and Si-O-Al [30].

The ATR/FTIR spectrum of pure EP601 shows the following characteristic absorption bands: at 3500 cm^−1^ (OH stretching vibrations); at 3057 and 3027 cm^−1^ (C-H stretching vibrations of the aromatic ring, asymmetric and symmetric, respectively); 2967cm^−1^ (asymmetric C-H stretching vibrations of the CH_3_ group); 2927cm^−1^ (asymmetric stretching vibrations C-H of the CH_2_ group); 2872cm^−1^ (symmetric C-H stretching vibrations of CH_2_ and CH_3_ groups); 1606, 1582, 1496 and 1455 cm^−1^ (C-C and C=C aromatic ring stretching vibrations); 1296 cm^−1^ (asymmetric C-H bending vibration in CH_2_); 1242 and 1033 cm^−1^ (C-O aromatic stretching vibrations, asymmetric and symmetric, respectively); 1183 cm^−1^ (C-O aliphatic stretching vibrations); 971, 915 cm^−1^ (characteristic vibrations of the epoxy ring); 828 cm^−1^ (deformation vibrations of the p-substituted benzene ring) [31].

The presence of free hydroxyl groups on the surface of both fillers causes these materials to be characterized by high polarity, and therefore hydrogen bonds occur between the OH groups and the associated water molecules. The introduction of such a polar filler to an epoxy resin, whose epoxy group is also polar in nature, will result in an increase in the number of hydrogen bonds between the surface OH groups of the filler and the oxygen atoms of the epoxy group, as well as an increase in the ether bond of the resin. The occurrence of such hydrogen bonds is described in detail by Maity et al. [31]. It should be noted that such newly formed hydrogen bonds will not introduce new peaks in the ATR/FTIR spectra.

The above analysis indicates that the addition of a filler to the polymer matrix (pure EP601 resin) will not significantly affect the ATR/FTIR spectra of the obtained composites. It results from the fact that the main absorption bands of the fillers largely overlap with the bands of pure resin, which makes them invisible in the spectra of the composites.

**Figure 6:**
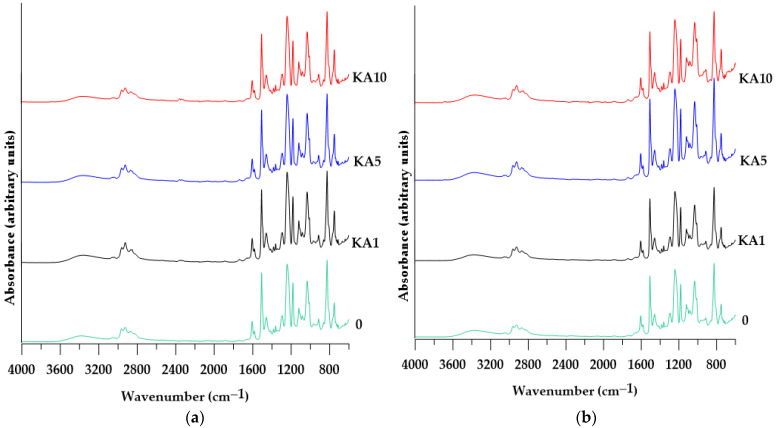

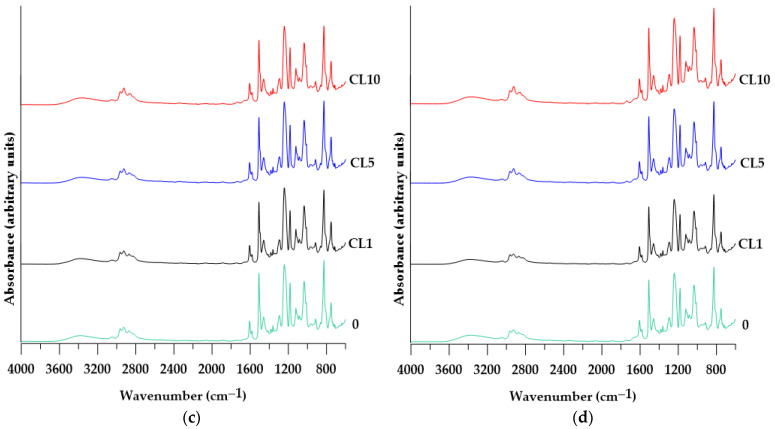
ATR/FTIR spectra of the materials obtained (**a**) KA before DMA analysis; (**b**) KA after DMA analysis; (**c**) CL before DMA analysis; (**d**) CL after DMA analysis.

The ATR/FTIR spectra of the materials obtained (see Figure 6) reveal characteristic absorption bands for the products of the cross-linking of the epoxy resin with TETA. These are the OH group stretching vibrations at about 3370 cm^−1^ and the corresponding C-O stretching vibrations in alcohols (absorption bands at about 1181 cm^−1^), the N-H bending vibrations at about 1507 cm^−1^, and the N-H wagging deformation vibrations at about 782 cm^−1^. In addition, the spectra show absorption bands in the range of 2853–2963 cm^−1^ corresponding to C-H stretching vibrations in the methyl and methylene groups. These vibrations are related to C-H bending vibrations at about 1458 cm^−1^. 

The presence of aromatic rings in the structure of the epoxy resin is confirmed by absorption bands at about 826 cm^−1^ (deformation vibrations of the *p*-substituted benzene ring), C=C ring stretching vibrations (at about 1606 and 1582 cm^−1^) and Ar-H stretching vibrations at about 3050 cm^−1^. In the range of 3050—3035 cm^−1,^ there are also asymmetric C-H stretching vibrations of the resin epoxy ring, while deformation vibrations of the entire epoxy ring in the ATR/FTIR spectra are characterized by absorption bands at about 915 cm^−1^ [4].

The FTIR spectra obtained after the DMA analysis did not show significant differences in the intensities of most bands, except for the band at about 915 cm^−1^, the intensity of which decreased slightly. This suggests a reduction in the number of free epoxide groups as a result of the cross-linking of the samples during heating.

### 3.2. DSC Analysis

**Figure 7:**
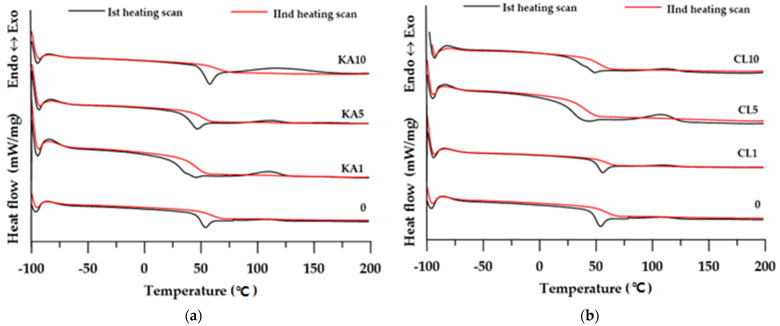
DSC curves of the prepared materials: (**a**) KA-based composites; (**b**) CL-based composites.

The changes the in the physical transformation of the materials obtained were determined by DSC analysis. The numerical data of the analyses are presented in Table 2, while the shapes of the DSC curves are presented in Figure 7. 

In the DSC curves from the first heating cycle (see Figure 7a), two energy effects are visible for all materials. The first one, in the range of 20–60 °C, is associated with the glassy transformation of the materials tested. The determined T_g_ values are in the range of 32–54 °C. Analyzing the influence of the filler addition on the T_g_ value, it is difficult to observe clear dependencies. In the series of materials based on kaolinite, the T_g_ of the materials increases with the increase in the amount of filler, while in the series with clinoptilolite, there is no such relationship. The second energy effect visible in the DSC curves from the first heating, which is an exothermic effect, can be characterized as an effect related to the cross-linking of materials. This process took place at cross-linking temperatures (T_cur_) between 107 and 117 °C, and the associated heat release (Δ*H*) was in the range of between 3.16 and 18.33 J/g. The cross-linking process of the obtained materials in both series did not depend on the amount of filler in the sample.

The DSC curves from the second heating revealed only glass transitions (T_g_ in the range of 40–65 °C) which were higher than those determined in the first heating. This is consistent with the common knowledge that the cross-linking process shifts the glass-transition temperatures of the material towards higher temperatures. 

As in the first heating cycle, in the series of materials with kaolinite, the T_g_ values increased with the increase in the amount of filler.

Comparing the effect of the type of filler (kaolinite and clinoptilolite) on the parameters determined by the DSC method, it can be concluded that the type of filler does not affect the thermal values of the composites obtained.

### 3.3. DMA Analysis

**Figure 8:**
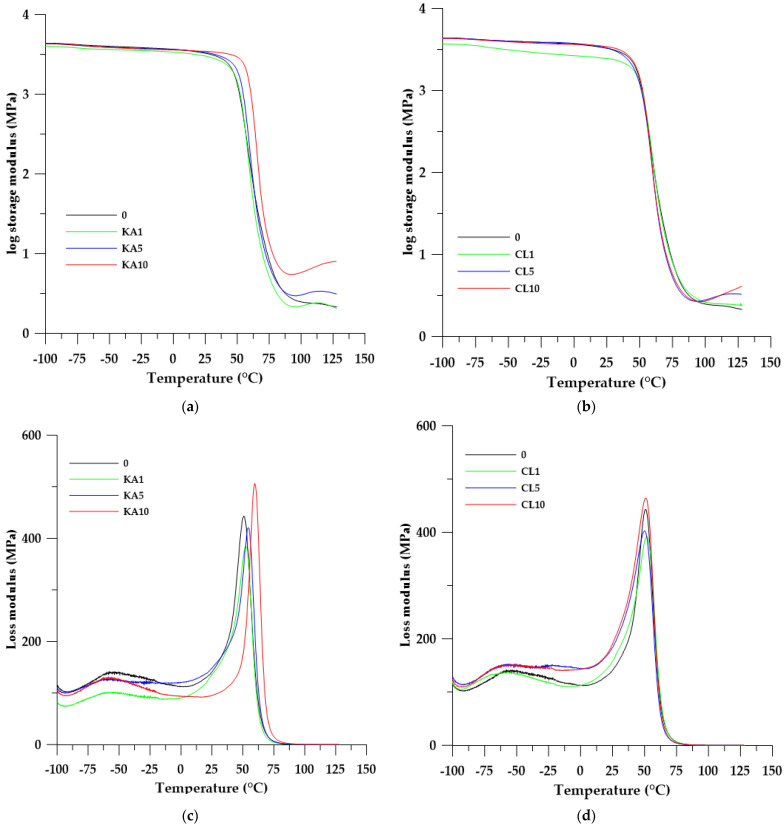

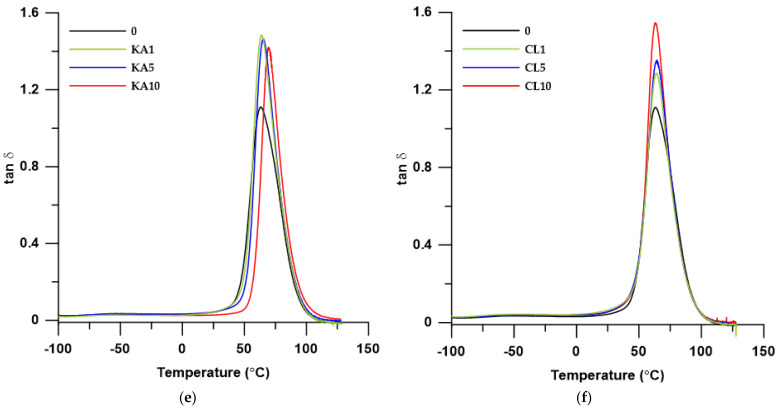
Temperature dependence of storage modulus (**a**,**b**) loss modulus (**c**,**d**) and tanδ (**e**,**f**) of the prepared materials.

To investigate the influence of the fillers on the viscoelastic properties of composites, dynamic mechanical thermal analysis was performed. Changes in storage modulus (*E′*), loss modulus (*E″*), and mechanical loss factor (tan δ) with temperature are shown in Figure 8, while DMA data are summarized in Table 3.

As can be seen from Table 3, the values of the storage modulus (*E′*_20_) characterizing the stiffness of the material increase with the increase in the filler content in the sample. Similar relationships can be observed for the loss modulus, which characterizes the elasticity of the tested materials. It is worth noting here that in the case of the loss modulus, the addition of 10% by weight of the filler increased the flexibility, while the other composites were characterized by lower *E″*_max_ values. 

The flexibility of the material is also characterized by the value of the damping factor (tan δ_max_). The higher its value, the less flexible the material is, which makes it less capable of damping vibrations. Analyzing the effect of the filler addition on this parameter, it can be seen that its addition increased the tan δ_max_ value with respect to the starting material. In the case of this parameter, there are clear differences in the type of filler used. In the series of composites with kaolinite, the tan δ_max_ values decreased as the filler content increased, while in the series with clinoptilolite this relationship was reversed. 

The addition of the filler in both series increased the homogeneity (FWHM values) of the composites obtained, compared with the material without the filler. This is interesting because, in general, the addition of any component to the polymer mixture reduces its homogeneity. In our case, the fillers used were to some extent bound to the polymer matrix through hydrogen bonds, as described in the section describing the ATR/FTIR spectra.

Interesting conclusions confirming the results of the DSC analysis can be drawn after analyzing the temperatures of the glass transitions. Using the DMA method, we determined the T_g_ values of materials from the maximum tan delta (tan *δ*_max_) and maximum loss modulus (*E″*_max_). T_g_ values from the loss modulus maximum were close to the T_g_ values determined by the DSC method, while values from tan δ_max_ were about 10–15 °C higher.

The shape of the storage modulus curves in Figure 8 correlates with the shape of the DSC curves from the first heating cycle. After the glass transition, which is shown by the inflection of the storage modulus curve, there is no flattening, but a rising of the line. This is due to the cross-linking of the sample during heating, which increases its stiffness. This effect is visible in both series of materials obtained.

However, when we trace the shape of the loss modulus and tan delta curves, two transformations can be observed. The first one (with maxima between −60 and −50 °C) is connected with the main chain movements of the polymeric matrix and local movements of polar groups. The second type of relaxation (called primary relaxation) with maxima between 25 and 70 °C is related to the glass transition of the polymer. The intensity of the principal relaxation peak determines the material’s ability to dampen (absorb) vibrations; the smaller its value, the greater the material’s damping capacity. 

**Figure 9:**
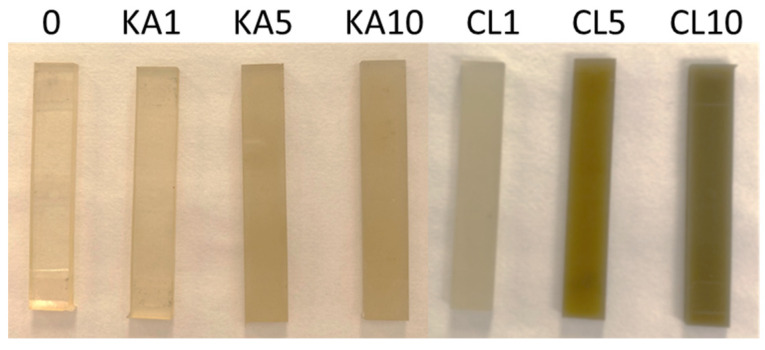
Samples after DMA analysis.

In Figure 9, the photos of the cut samples after DMA analysis are presented. As can be seen in the photo, heating the samples to 127 °C did not damage the samples.

### 3.4. Mechanical Properties

**Figure 10:**
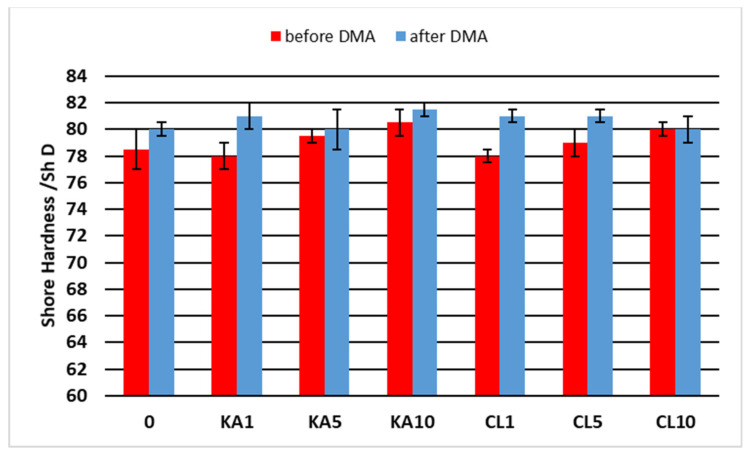
The hardness of the obtained materials.

For the obtained materials, the hardness was tested before and after the DMA analysis, and the results are presented in Figure 10.

The materials obtained were characterized by Shore hardness values before DMA analysis in the range of 78.5–80.5 ShD, and after DMA analysis in the range of 80.0–81.5 ShD. As Figure 10 shows, the hardness of the materials increased with the increase in the content of the filler, and the type of filler did not affect the differences in hardness. In all cases, the DMA analysis increased the hardness of the samples. This is consistent with earlier observations from DSC and DMA analyses, which showed that after heating the samples, their cross-linking occurred.

The increase in hardness of the composites after DMA analysis may result from several factors, the occurrence of which was dependent on the heating of the samples. As already mentioned, when the samples were heated above 110 °C, additional chemical cross-linking took place, which resulted in the opening of the epoxide ring and the formation of new hydroxyl groups. Apart from cross-linking, the water molecules associated on the surface of the filler were also released during heating, which increased the number of free hydroxyl groups. This made the surface more polar. A greater number of hydroxyl groups on the surface of the filler and in the polymer matrix resulted in an increase in the amount of hydrogen bonds between the phases and thus in the hardness as well as stiffness of the obtained composites.

In Table 4 the bending and tensile strain data are presented.

The DIC method also enables the observation of deformation maps, allowing for a visual determination of which zone experiences the highest deformation and where the material is under the most stress. Table 5 presents EpsX deformation maps at the stage just before sample destruction. One sample for each tested type was selected.

**Figure 11:**
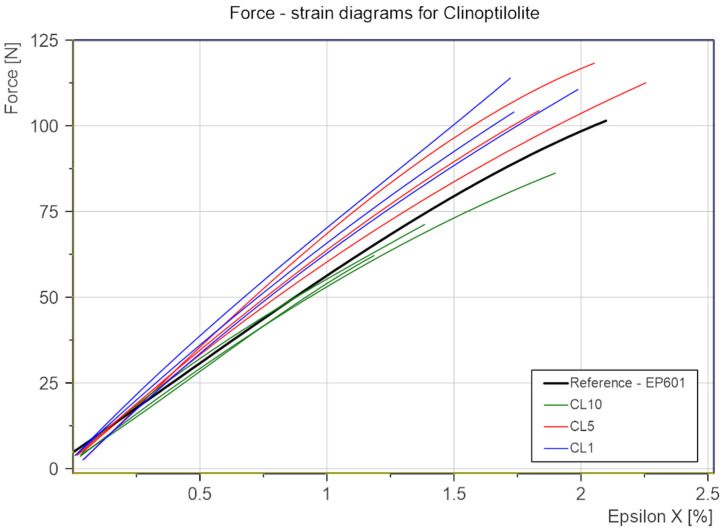
Bending force-tensile strains for CL.

**Figure 12:**
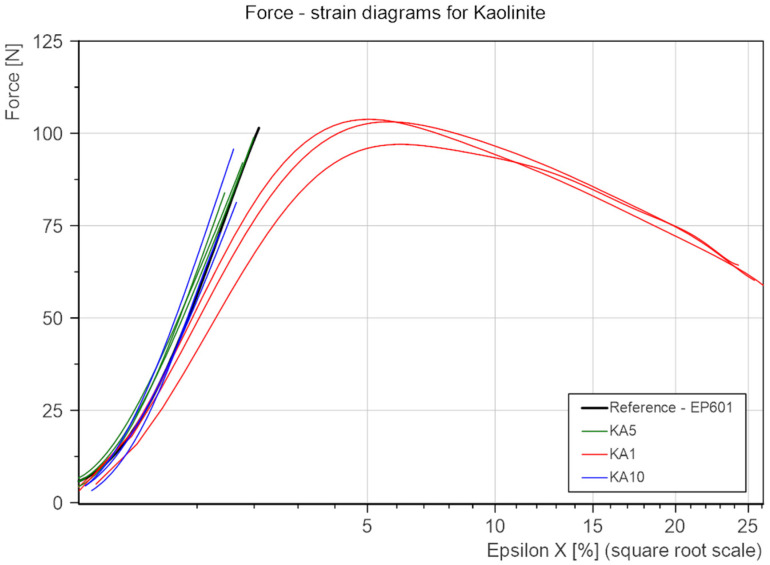
Bending force-tensile strains for KA.

To compare the influence on the strength properties of the percentage of added KA and CL. the results were collated into two groups (Figure 11 and Figure 12). Due to the insignificant difference in the cross-sectional area of all samples (the standard deviation in this range is 0.88 mm^2^ compared to the mean cross-sectional area of all samples at the level of 35.71 mm^2^), the analysis of bending force vs. deformation was chosen instead of bending stress vs. deformation.

**Figure 13:**
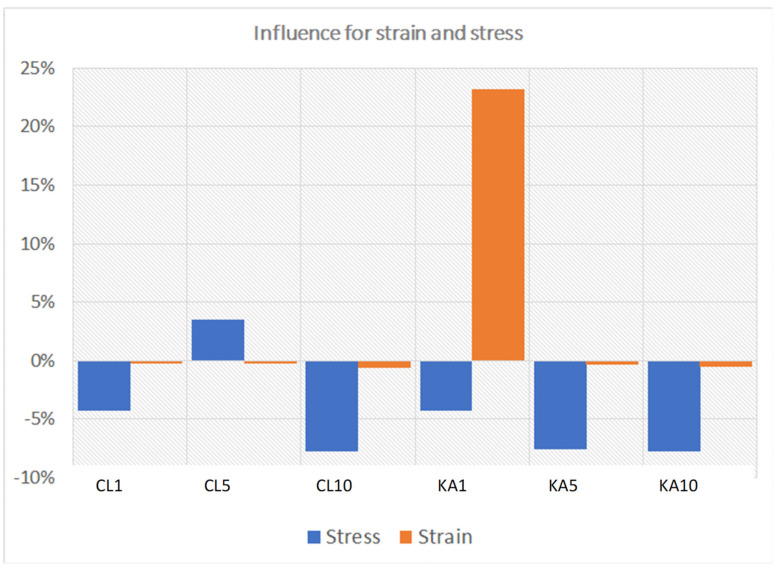
Percentage influence on improvement/deterioration of strength properties.

In the case of kaolinite (Figure 11), the addition at the level of 5% and 10% slightly worsens both properties analyzed. However, in the case of the amount at the level of 1%, material plasticization occurs, which leads to the possibility of carrying deformations over 16 times greater than the reference material (25% deformation compared to 1.5% deformation). This is visually presented in Figure 13.

Level 0% in Figure 13 was taken as the reference material value. A positive percentage change indicates an improvement in properties, while a negative change indicates a deterioration in properties.

### 3.5. SEM Analysis

**Figure 14:**
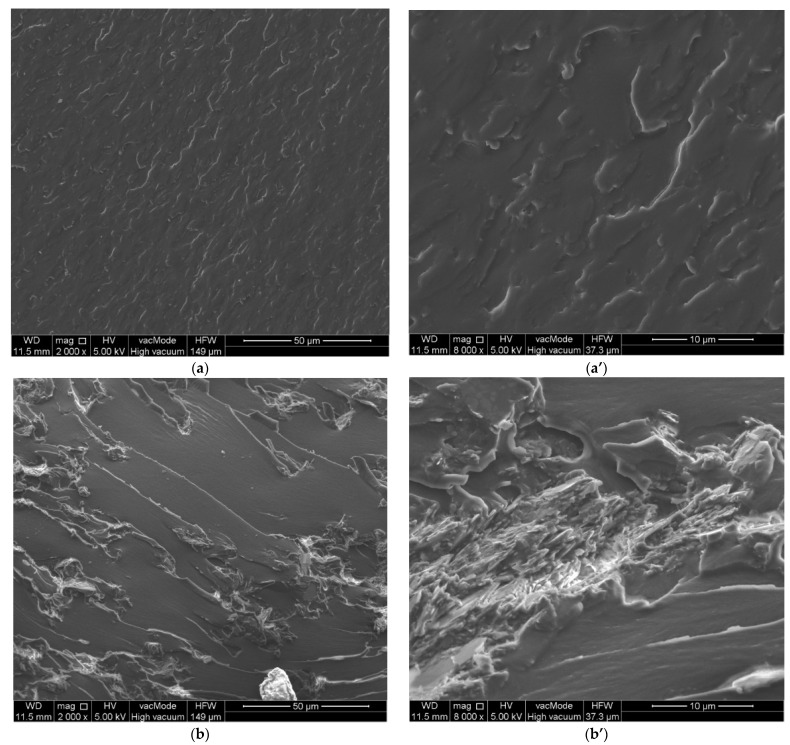

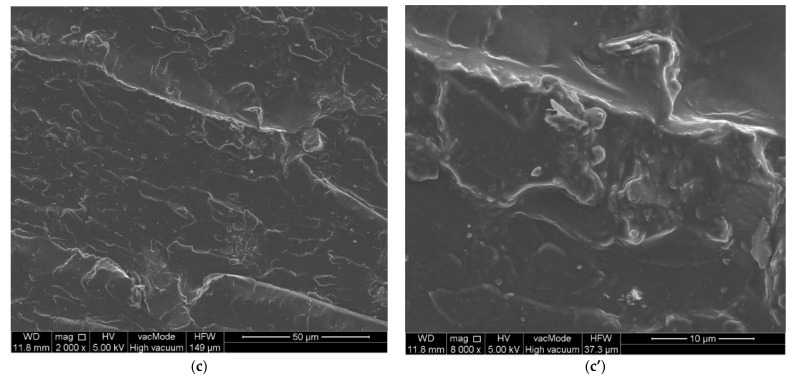
Microphotographs of the structure of the composites: (**a**,**a’**) sample O (left side before strength tests, right side after strength tests); (**b**,**b’**) KA10; (**c**,**c’**) CL10.

In Figure 14 the SEM images of the composites being studied before and after mechanical tests are presented.

Studies of the microstructure of the composites obtained using scanning electron microscopy (SEM) show the different nature of the two mineral additives. The surfaces of the reference sample are characterized by a homogeneous internal structure (Figure 14a). The addition of both kaolinite and clinoptilolite causes the distinct appearance of thin overlapping layers in the microstructure of the materials tested. The structure of these composites becomes less homogeneous, and pear-shaped aggregates of the applied additives are visible (Figure 14b,c). This is particularly evident in composites whose composition was modified by the addition of kaolinite. Analyzing the surfaces obtained as a result of destructive forces in composites containing in their composition the addition of kaolinite or clinoptilolite, it is clear that the process of destruction took place on the mineral aggregates present in the structure of the tested materials (Figure 14b’,c’).

## 4. Conclusions

During the crosslinking reaction, the new composites with advantageous thermal and mechanical properties are obtained from the epoxy resin (Epidian 601) with triethylenetetramine and inorganic mineral fillers. The FTIR analysis confirms the opening of the epoxy ring reaction, as the decrease of 915 cm^−1^ signal in the composite spectra is visible. DSC analysis shows that the type of filler (kaolinite and clinoptilolite) does not affect the thermal values of the composites obtained. The T_g_ values of the materials obtained are in the range of 30–54 °C, which is typical for crosslinked epoxy resins. 

The addition of the filler to polymer matrices caused an increase in the homogeneity as well as stiffness, as indicated by dynamical thermal analysis. As can be seen from DSC and DMA analysis, upon heating the materials obtained became harder. It indicates the crosslinking effect on the samples after increasing the temperature.

By analyzing the effect of the percentage addition of clinoptilolite on strength properties, it can be unequivocally stated that for the added value of 10% the properties deteriorated. Both the maximum force carried by the sample in the bending test and the maximum tensile strains in the lower layer of the sample are lower than those of the reference material. However, at addition values of 1% and 5% there is a slight improvement in the maximum force value and a slight decrease in the strain values for 1% clinoptilolite, and strain values for 5% clinoptilolite are similar to those in the reference material.

The confirmed appropriate thermal, thermo-mechanical, and mechanical properties of the materials obtained indicate the possibility of their application f.ex. in the building industry. 

## Figures and Tables

**Table 1:** Amounts of chemicals used for the synthesis of composites.

Composite	EP601	TETA [g]	KA	CL
0	15	1.5	0	0
KA1	15	1.5	0.165	0
KA5	15	1.5	0.825	0
KA10	15	1.5	1.650	0
CL1	15	1.5	0	0.165
CL5	15	1.5	0	0.825
CL10	15	1.5	0	1.650

**Table 2:** DSC data of the prepared materials.

Sample	T_g_ [°C]		T_cur_ [°C]		Δ*H* [J/g]	
	I ^a^	II ^b^	I ^a^	II ^b^	I ^a^	II ^b^
0	50	59	107	-	3.65	-
KA1	32	47	110	-	4.78	-
KA5	43	50	112	-	3.37	-
KA10	54	65	117	-	18.33	-
CL1	53	58	109	-	3.16	-
CL5	31	40	107	-	7.10	-
CL10	45	53	111	-	3.39	-

^a, b^ first and second heating scan, respectively.

**Table 3:** DMA results of the prepared materials.

Sample	*E′*_20_ (GPa)	*E″*_max_ (°C)	*E″*_max_ (MPa)	tan *δ*_max_ *(*°C)	tan *δ*_max_	FWHM (°C)
0	3.01	50.9	443.1	63.3	1.10	26.73
KA1	3.11	52.3	384.6	64.1	1.47	21.44
KA5	3.43	54.3	420.7	65.0	1.44	20.20
KA10	3.48	59.4	505.7	69.7	1.40	17.91
CL1	3.14	51.7	391.9	63.8	1.27	21.89
CL5	3.43	49.8	402.5	64.5	1.34	21.65
CL10	3.50	50.9	464.1	63.2	1.52	19.60

**Table 4:** Bending and tensile strain results.

Spec. Name	Material Name	Strength for Bending (Zwick) [MPa]	Average Strength for Material [MPa]	Maximum Deflection (ARAMIS) [mm]	Average Deflection per Material [mm]	Maximum Tensile Strain EpsX (ARAMIS) [%]	Average from Maximum Strain per Material [%]
1.3	CL5	73.6	81.0	3.62	3.62	1.83	1.83
1.2		87.4		4.12		2.05	
1.4		81.9		4.16		2.25	
2.2	KA1	70.0	73.2	15.98	17.74	24.28	25.26
2.3		74.7		18.01		25.45	
2.4		74.9		19.22		26.06	
3.1	CL10	51.0	58.3	2.39	3.00	1.18	1.49
3.2		68.6		3.73		1.90	
3.3		55.3		2.88		1.38	
4.2	0	19.4	48.8	8.92	8.51	1.08	6.61
4.3		77.5		3.97		2.10	
4.4		49.5		12.65		16.64	
5.1	KA5	62.3	69.9	3.01	3.41	1.44	1.73
5.2		72.7		3.56		1.77	
5.3		74.8		3.67		1.99	
6.1	CL1	83.2	88.3	3.53	3.74	1.74	1.82
6.2		92.3		3.85		1.72	
6.3		89.4		3.84		1.99	
7.1	KA10	62.8	69.7	2.63	2.90	1.36	1.54
7.2		79.9		3.17		1.60	
7.3		66.3		2.90		1.65	

**Table 5:** EpsX deformation maps.

Material Name	Spec. Name	EpsX Strain Maps View and the Scale. 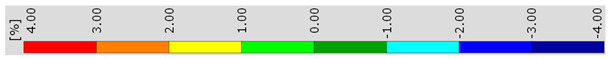
0	4.3	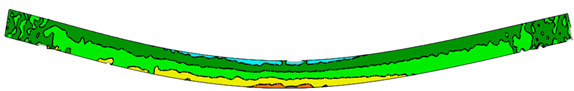
CL1	6.3	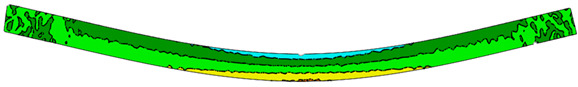
KA5	1.4	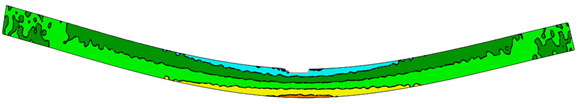
CL10	3.3	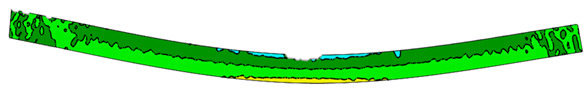
KA1	2.3	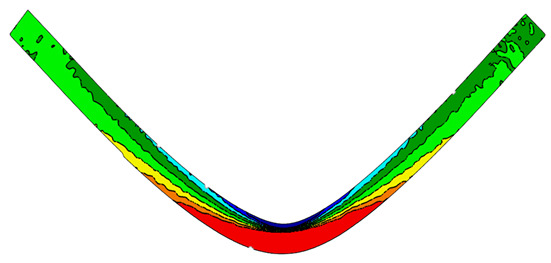
KA5	5.1	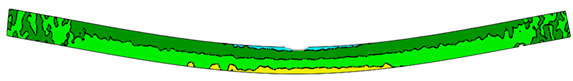
KA10	7.3	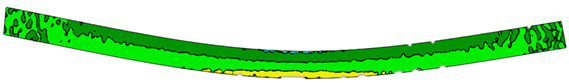

## Data Availability

All data are available from the corresponding author upon reasonable request.
